# Performance of 2 Finger-Stick Blood Tests to Triage Adults With Symptoms of Pulmonary Tuberculosis: A Prospective Multisite Diagnostic Accuracy Study

**DOI:** 10.1093/cid/ciaf105

**Published:** 2025-04-16

**Authors:** Jayne S Sutherland, Gian D van der Spuy, Jane A Shaw, Tracy Richardson, Elisa M Tjon Kon Fat, Awa Gindeh, Olumuyiwa Owolabi, Nguyen Thuy Thuong Thuong, Le Hong Van, Nguyen Hoang Van, Dang Thi Thanh Thao, Harriet Mayanja-Kizza, Mary Nsereko, AnnRitah Namuganga, Sophie Nalukwago, John Belisle, Emmanuel Moreau, Adam Penn-Nicholson, Guy Thwaites, Jill Winter, Hazel M Dockrell, Thomas J Scriba, Kim Stanley, Bronwyn Smith, Novel N Chegou, Stephanus T Malherbe, Annemieke Geluk, Paul Corstjens, Gerhard Walzl, the TrENDx-TB consortium, Jayne S Sutherland, Olumuyiwa Owolabi, Amie Secka, Bintou Njai, Abdou K Sillah, Georgetta K Daffeh, A Gindeh, Amadou Barry, Momodou Rashid, Joseph Mendy, Binta Sarr, Abi-Janet Riley, Alhaji Jobe, Monica Davies, Kairaba Kanyi, Momodou Jallow, Salieu Barry, Ousainou Cham, Gerhard Walzl, Stephanus T Malherbe, Bronwyn Smith, Gian D van der Spuy, Kim Stanley, Jane A Shaw, Alicia Chetram, Tracy Richardson, Bernadine Fransman, Isaac Johnson, Marika Finn, Andriette Hiemstra, Novel N Chegou, Helena Kuivaniemi, Gerard Tromp, Susanne Tonsing, Elizma Smit, Balie Carstens, Harriet Mayanja-Kizza, Mary Nsereko, AnnRitah Namuganga, Sophie Nalukwago, Joseph Akol, Saidah Menya, Veronica Kizza, Yusuf Kironde, Deborah Banturaki, Immaculate Nahereza, Simon Okiror, Immaculate Kemigisha, Paul Mutumba, Henry Ojiambo, Lilian Murungi, Joan Nassuna, Gladys Mpalanyi, Michael Odie, Guy Thwaites, Nguyen Thuy Thuong Thuong, Van Le, Son Vo Thanh, Hau Nguyen Thi, Ha Vu Thi Ngoc, Ngoc Le Hong, John Belisle, Karen Dobos, Hazel M Dockrell, Thomas J Scriba, Mark Hatherill, Kate Hadley, Justin Shenje, Stanley Kimbung, Humphrey Mulenga, Rachel Oelofse, Nicole Bilek, Elma van Rooyen, Simba Mabwe, Paul Corstjens, A Geluk, Elisa M Tjon Kon Fat, Louise Pierneef, Anouk van Hooij, Jill Winter, Morten Ruhwald, Emmanuel Moreau, Adam Penn-Nicholson, Claudia Schacht, Julia Büech

**Affiliations:** Vaccines and Immunity Theme, MRC Unit The Gambia at the London School of Hygiene and Tropical Medicine, Banjul, The Gambia; Department of Infection Biology, Faculty of Infectious and Tropical Diseases, London School of Hygiene and Tropical Medicine, London, United Kingdom; DSI-NRF Centre of Excellence for Biomedical Tuberculosis Research, South African Medical Research Council Centre for Tuberculosis Research, Division of Immunology, Department of Biomedical Sciences, Faculty of Medicine and Health Sciences, Stellenbosch University, Cape Town, South Africa; DSI-NRF Centre of Excellence for Biomedical Tuberculosis Research, South African Medical Research Council Centre for Tuberculosis Research, Division of Immunology, Department of Biomedical Sciences, Faculty of Medicine and Health Sciences, Stellenbosch University, Cape Town, South Africa; DSI-NRF Centre of Excellence for Biomedical Tuberculosis Research, South African Medical Research Council Centre for Tuberculosis Research, Division of Immunology, Department of Biomedical Sciences, Faculty of Medicine and Health Sciences, Stellenbosch University, Cape Town, South Africa; Department of Cell and Chemical Biology, Leiden University Medical Center, Leiden, The Netherlands; Vaccines and Immunity Theme, MRC Unit The Gambia at the London School of Hygiene and Tropical Medicine, Banjul, The Gambia; Vaccines and Immunity Theme, MRC Unit The Gambia at the London School of Hygiene and Tropical Medicine, Banjul, The Gambia; Oxford University Clinical Research Unit, Ho Chi Minh City, Vietnam; Centre for Tropical Medicine and Global Health, Nuffield Department of Medicine, University of Oxford, Oxford, United Kingdom; Oxford University Clinical Research Unit, Ho Chi Minh City, Vietnam; Centre for Tropical Medicine and Global Health, Nuffield Department of Medicine, University of Oxford, Oxford, United Kingdom; Oxford University Clinical Research Unit, Ho Chi Minh City, Vietnam; Centre for Tropical Medicine and Global Health, Nuffield Department of Medicine, University of Oxford, Oxford, United Kingdom; Oxford University Clinical Research Unit, Ho Chi Minh City, Vietnam; Centre for Tropical Medicine and Global Health, Nuffield Department of Medicine, University of Oxford, Oxford, United Kingdom; Department of Medicine, Makerere University, Kampala, Uganda; Department of Medicine, Makerere University, Kampala, Uganda; Department of Medicine, Makerere University, Kampala, Uganda; Department of Medicine, Makerere University, Kampala, Uganda; Mycobacteria Research Laboratories, Department of Microbiology, Immunology and Pathology, Colorado State University, Fort Collins, Colorado, USA; FIND, Geneva, Switzerland; FIND, Geneva, Switzerland; Oxford University Clinical Research Unit, Ho Chi Minh City, Vietnam; Centre for Tropical Medicine and Global Health, Nuffield Department of Medicine, University of Oxford, Oxford, United Kingdom; Catalysis Foundation, Berkeley, California, USA; Department of Infection Biology, Faculty of Infectious and Tropical Diseases, London School of Hygiene and Tropical Medicine, London, United Kingdom; Division of Immunology, Department of Pathology, South African Tuberculosis Vaccine Initiative, Institute of Infectious Disease and Molecular Medicine, University of Cape Town, Cape Town, South Africa; DSI-NRF Centre of Excellence for Biomedical Tuberculosis Research, South African Medical Research Council Centre for Tuberculosis Research, Division of Immunology, Department of Biomedical Sciences, Faculty of Medicine and Health Sciences, Stellenbosch University, Cape Town, South Africa; DSI-NRF Centre of Excellence for Biomedical Tuberculosis Research, South African Medical Research Council Centre for Tuberculosis Research, Division of Immunology, Department of Biomedical Sciences, Faculty of Medicine and Health Sciences, Stellenbosch University, Cape Town, South Africa; DSI-NRF Centre of Excellence for Biomedical Tuberculosis Research, South African Medical Research Council Centre for Tuberculosis Research, Division of Immunology, Department of Biomedical Sciences, Faculty of Medicine and Health Sciences, Stellenbosch University, Cape Town, South Africa; DSI-NRF Centre of Excellence for Biomedical Tuberculosis Research, South African Medical Research Council Centre for Tuberculosis Research, Division of Immunology, Department of Biomedical Sciences, Faculty of Medicine and Health Sciences, Stellenbosch University, Cape Town, South Africa; Department of Infectious Diseases, Leiden University Medical Center, Leiden, The Netherlands; Department of Infectious Diseases, Leiden University Medical Center, Leiden, The Netherlands; DSI-NRF Centre of Excellence for Biomedical Tuberculosis Research, South African Medical Research Council Centre for Tuberculosis Research, Division of Immunology, Department of Biomedical Sciences, Faculty of Medicine and Health Sciences, Stellenbosch University, Cape Town, South Africa

**Keywords:** Biomarkers, tuberculosis, triage, point-of-care, diagnosis

## Abstract

**Background:**

Non–sputum-based, point-of-care triage tests for pulmonary tuberculosis could enhance tuberculosis diagnostic programs. We assessed the diagnostic accuracy of 2 finger-stick blood tests: the Cepheid 3 gene host-response cartridge (Xpert-HR), which measures 3 host messenger RNA transcripts, and the 3-host protein multibiomarker test (MBT).

**Methods:**

We performed a prospective diagnostic accuracy study of consecutive participants with symptoms compatible with pulmonary tuberculosis in The Gambia, South Africa, Uganda, and Vietnam. A composite reference standard for active pulmonary tuberculosis incorporated chest radiography, symptom resolution, and sputum microbiological test results. A training-test set approach was used to evaluate test cutoff specificities at 90% sensitivity.

**Results:**

Between 1 November 2020 and 1 May 2023, we screened 1262 participants aged 12–70 years with cough lasting >2 weeks and another symptom suggestive of tuberculosis. Of those who were classifiable by reference tests, 1154 participants had evaluable Xpert-HR results and 961 had evaluable MBT results. Xpert-HR had an area under the receiver operating characteristic (AUROC) curve of 0.92 at a cutoff of −1.275 or below, with a sensitivity of 92.8%, specificity of 62.5%, positive predictive value of 47.9%, and negative predictive value of 95.9%. The MBT had an AUROC of 0.91 at a cutoff of ≥0.42, with a sensitivity of 91.4%, specificity of 73.2%, positive predictive value of 52.0%, and negative predictive value of 96.4%.

**Conclusions:**

Our results show that both Xpert-HR and the MBT are promising non–sputum-based point-of-care tests. The MBT met the World Health Organization target product profile for a triage test, which suggests it should be further developed.

## Teaser

Two finger-stick blood tests to triage people with symptoms of pulmonary tuberculosis by measuring 3 host proteins (multibiomarker test) or 3 host RNA transcripts (Cepheid Xpert-HR) yielded sensitivities >90% in patients from The Gambia, South Africa, Uganda, and Vietnam.

## Graphical Abstract

**Graphical Abstract:**
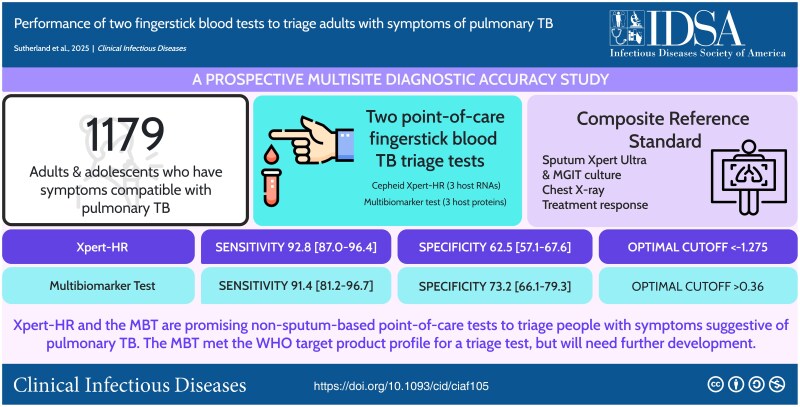
A graphic depicting the study population, the diagnostic tests being assessed, the reference standard, and the main results. This graphical abstract is also available at Tidbit: https://tidbitapp.io/institutional-portal/clinical-infectious-diseases/tidbits/performance-of-two-fingerstick-blood-tests-to-triage-adults-with-symptoms-of-pulmonary-tb-a-prospective-multisite-diagnostic-accuracy-study/update

A non–sputum-based point-of-care triage test for pulmonary tuberculosis is one of the highest priorities for development in the tuberculosis diagnostics pipeline. Of the estimated 10.8 million cases of tuberculosis in 2023, approximately 2.5 million went undiagnosed, partly due to the lack of adequate diagnostic tools [1]. The diagnosis of pulmonary tuberculosis depends on the detection of *Mycobacterium tuberculosis* in respiratory samples, but these assays come with a high cost and infrastructure requirements. A triage test at the point of care would address this problem by accurately distinguishing between people who do and those who do not need further confirmatory testing when they present with symptoms compatible with tuberculosis.

The Cepheid 3 gene host-response cartridge (also known as the Xpert MTB Host Response assay, or Xpert-HR) is based on detection of a 3-gene signature (*GBP5*, *DUSP3*, and *KLF2*) identified by Sweeney et al [2], which has been translated to a near-point-of-care, finger-stick blood test with automatic calculation of a TB score [2]. This score reached the World Health Organization (WHO) target product profile (TPP) for a non–sputum-based triage test, with a sensitivity >90% and a specificity of >70% across 3 independent prospective cohorts [3]. We have previously published interim results in which the Xpert-HR performed well with, sensitivity and specificity of 87% and 94%, respectively [4]. However, findings from a cohort of 1499 adults showed good classification of tuberculosis compared to other respiratory diseases (area under the receiver operating characteristic curve [AUROC], 0.89; sensitivity, 90%) but the specificity was only 63% [5], and a pan-African study of 639 children aged <15 years reported a sensitivity of 60% and a specificity of 90% [6].

The detection of multiple host protein markers also holds promise for a point-of-care test [7]. We have previously identified a 7-host protein serum signature for tuberculosis from >700 participants across 5 African countries using exploratory proteomics [8]. This signature has since been refined to 3 markers, including serum amyloid A, C-reactive protein, and interferon (IFN) γ–inducible protein 10, which was assessed in a previous study (clinical trial NCT03350048) and translated to a lateral flow test using up-converting reporter particle technology, by members of the TrENDx-TB consortium [9]. The aim of the current study was to perform a prospective multicountry head-to-head comparison of these 2 quantitative point-of-care tests to triage adults and adolescents presenting with respiratory symptoms suggestive of pulmonary tuberculosis.

## METHODS

### Study Design and Participants

This was a cross-sectional diagnostic accuracy study performed in The Gambia, South Africa, Uganda and Vietnam. Consecutive participants aged 12–70 years with symptoms suggestive of pulmonary tuberculosis were recruited when they presented to primary health facilities or hospitals. Inclusion criteria were a cough for >2 weeks and at least one other symptom suggestive of tuberculosis (ie, weight loss, hemoptysis, night sweats, fever), in line with the WHO W4SS symptom screen [10]. Potential participants were excluded if they were on tuberculosis-preventive therapy, were taking immunosuppressive treatment, were pregnant or breastfeeding, or if their hemoglobin level was <9 g/L. Written informed consent was obtained before sample collection. For participants aged 12–18 years, parent/guardian consent was obtained alongside participant assent. Local ethical approval was obtained at each participating site. The trial was registered at ClinicalTrials.gov with the (no. NCT04232618). The full protocol is published under a Creative Commons Attribution 4.0 International License [11].

### Procedures

A detailed medical history was taken on enrollment, and all participants were tested for human immunodeficiency virus (HIV) with rapid finger-stick blood test and a confirmatory enzyme-linked immunosorbent assay if results were positive and for diabetes with finger-stick blood glucose and confirmatory Hemoglobin A1c. Nasopharyngeal swab samples were tested with multiplex 26-pathogen polymerase chain reaction (PCR [Allplex; Seegene]) as well as an in-house severe acute respiratory syndrome coronavirus (SARS-CoV-2) PCR assay or GeneXpert SARS-CoV-2 Xpert Xpress cartridges (Cepheid). A subset was also tested with anti–SARS-CoV-2 antibody tests (see the Supplementary methods). A chest radiograph was reported independently on a standardized reporting tool by 2 blinded medical officers, with a third reading by a specialist in the absence of consensus.

Sputum was tested with BACTEC Mycobacterial Growth Indicator Tube liquid culture (Becton Dickinson), Xpert Ultra (Cepheid), and smear microscopy with Auramine staining for acid-fast bacilli. If tuberculosis was not diagnosed from reference tests, the participant was brought back for repeated clinical evaluation 8 weeks after enrollment to establish the alternative diagnosis. Drug-resistant pulmonary tuberculosis and extrapulmonary tuberculosis without evidence of pulmonary infection were excluded when these results became available. IFN γ release assays and tuberculin skin testing were not performed as positivity rates in African settings are very high and the test would therefore not contribute to the classification of participants with respiratory symptoms [8, 12].

Index tests and reference tests were performed at the same visit. For the index tests, 200 µL of capillary blood was collected by finger-stick with a Minivette containing an anticoagulant (ethylenediaminetetraacetic acid [EDTA]; Becton Dickinson), transferred into an EDTA-microtainer (Becton Dickinson), and inverted to mix. Next, 100 µL of the sample was added to the Xpert-HR cartridge (donated by Cepheid) and loaded into the GeneXpert machine. Cycle threshold (Ct) values for individual genes (*GBP5*, *DUSP3*, *TBP*, *KLF2*) were obtained together with a TB score automatically calculated with the following formula: (*GBP5* Ct + *DUSP3* Ct)/2 − *TBP* Ct [4]. The manufacturer currently recommends that *TBP* be used in the place of *KLF2* from the first iteration of the TB score, to improve stability and performance. No prespecified cutoff value was provided by the manufacturer.

For the multibiomarker test (MBT), a further 20 μL of finger-stick blood was collected using disposable Minivette collection tubes (heparin coated; Sarstedt) and directly mixed with 480 μL of high-salt finger-stick buffer (100 mmol/L Tris pH 8.0, 270 mmol/L sodium chloride, 1% [vol/vol] Triton X-100, and 0.5% [wt/vol] casein) [7,9]. Next, 100 µL of the lysed blood solution was added to a disposable microwell, after which lateral flow strips (serum amyloid A, C-reactive protein, and IFN γ–inducible protein 10) were added. Lateral flow was continued until strips were dry, after which they were analyzed with the ESEQuant (LR3 version) reader adapted for the up-converting reporter particle label (DIALUNOX). Test results were displayed as the ratio of test to flow-control signal. Further details of the MBT manufacturing are available in the Supplementary methods.

### Analysis

The primary outcome was the diagnostic accuracy of the Xpert-HR and MBT compared with a composite reference standard (CRS) for active pulmonary tuberculosis, as shown in Table 1. Secondary outcomes included comparing performance between sites, assessing the effect of other respiratory pathogens on the test performance, and assessing test performance against Xpert Ultra alone.

**Table 1.** Composite Reference Standard Case Definitions in Participants Presenting With Symptoms Suggestive of Pulmonary Tuberculosis

Tuberculosis Category	Study Definition
Definite tuberculosis (tuberculosis positive)	Sputum culture positive for *Mycobacterium tuberculosis* *or* Xpert Ultra positive higher than ‘trace’ *or* Xpert Ultra trace positive, no previous tuberculosis, and smear positive
Probable tuberculosis (tuberculosis positive)	Chest radiograph in keeping with active tuberculosis or sputum smear positive *and* Good response to tuberculosis treatment, with no alternate diagnosis made
Possible tuberculosis	All sputum tests negative *and* Chest radiograph not in keeping with active tuberculosis but good response to tuberculosis treatment and no alternate diagnosis was made *or* Chest radiograph in keeping with active tuberculosis, no alternate diagnosis made, but tuberculosis treatment response unknown (not treated)
Tuberculosis negative	All sputum tests negative, chest radiograph not in keeping with active tuberculosis, symptoms resolved without tuberculosis treatment or alternate diagnosis was made *or* Sputum smear positive, Xpert Ultra trace positive, or any sputum test transiently positive (negative at repeated test), chest radiograph not in keeping with active tuberculosis, symptoms resolved without treatment or alternate diagnosis was made

All analysis was done using R software (version 4.1) on a server running Ubuntu Linux (22.04) [13]. For the Xpert-HR test, the dataset was divided into training and test sets at a ratio of 75:25. The training data were used to generate an ROC curve based on the predefined TB score, and the threshold score at 90% sensitivity was recorded. This threshold was then used to determine the performance on the test set. For the MBT, the dataset was divided into training and test sets at a ratio of 60:40. An elastic-net logistic regression model was trained on the training data using the MLR3 machine-learning framework [14]. Before training, the data were scaled and then upsampled using the SMOTE algorithm to balance the outcome groups. The model hyperparameters were tuned using bayesian optimization over repeated 3-fold cross-validation to maximize the AUROC. The trained model was then used to generate an ROC curve, and the threshold probability at 90% sensitivity was recorded. The model was then used to predict outcome probabilities on the test data and the training threshold was used to determine the performance.

For both tests, 95% bias-corrected and accelerated confidence intervals (CIs) for the performance metrics were calculated using bootstrapping (5000 replicates). To assess the impact of other respiratory pathogens on test performance, the models were run with all participants grouped by the presence or absence of a virus or bacteria on a nasopharyngeal swab sample PCR test.

## RESULTS

**Figure 1.**
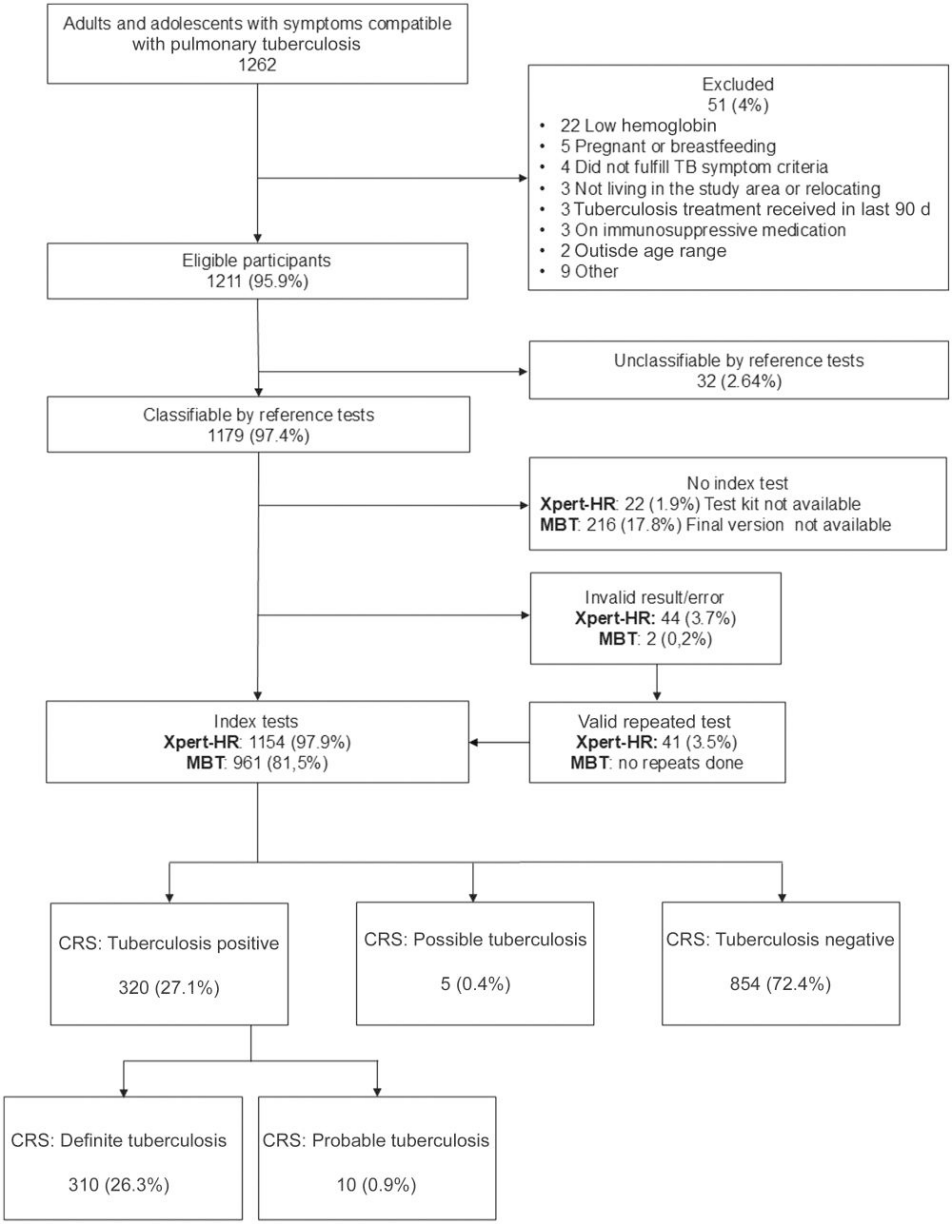
STARD diagram of participants in study. Details of the composite reference standard are provided in Table 1. Abbreviations: CRS, composite reference standard; MBT, multibiomarker test; Xpert-HR, Xpert MTB Host Response assay (Cepheid).

We screened 1262 participants with symptoms suggestive of tuberculosis between 1 November 2020 and 1 May 2023, of whom 51 (4%) were excluded (Figure 1). Of the remaining 1211 participants, 32 (2.6%) were unclassifiable by the CRS. Five (0.4%) were classified as having possible tuberculosis and were not included in the main analysis. Of classifiable participants, 1154 (97.9%) were tested with the Xpert-HR and 961 (81.5%) with the final version of the MBT. The first 216 participants were tested with an earlier version of the MBT, with results used to refine the final version, and are not included in this analysis. The characteristics of the participants are shown in Table 2 (stratified by site in Supplementary Table 1).

**Table 2.** Participant Characteristics by Tuberculosis Outcome Classification

Characteristic	Participants by Tuberculosis Outcome, No. (%)^a^				
	All (n = 1211)	Tuberculosis Positive (n = 320)	Tuberculosis Negative (n = 854)	Possible Tuberculosis (n = 5)	Unclassifiable (n = 32)
Female sex	501 (41.4)	88 (27.5)	403 (47.2)	1 (20.0)	9 (28.1)
Age, median (IQR), y	34.4 (25.9–45.1)	32.1 (16.3–47.9)	34.8 (15.2–54.2)	46 (25.8–65.4)	37.6 (17.3–59.9)
BMI, median (IQR)^b^	20.1 (18–22.9)	18 (14.6–21.4)	20.9 (15.8–26)	21.8 (16–25)	18.2 (14.1–22.3)
Prior tuberculosis	185 (16.5)	29 (9.1)	143 (16.7)	3 (60.0)	10 (31.3)
Tuberculosis features					
Cough for >2 wk	1211 (100.0)	320 (100.0)	854 (100.0)	5 (100.0)	32 (100.0)
Night sweats	515 (42.5)	146 (45.6)	354 (41.5)	3 (60.0)	12 (37.5)
Fever	507 (41.9)	172 (53.8)	319 (37.4)	0	16 (50.0)
Loss of weight	593 (48.9)	208 (65.0)	364 (42.6)	3 (60.0)	19 (59.4)
Loss of appetite	484 (39.9)	170 (53.1)	298 (34.9)	3 (60.0)	13 (40.6)
Chest pain	758 (62.6)	216 (67.5)	517 (60.5)	2 (66.7)	23 (71.9)
Chest radiographic cavities	226 (20.2)	176 (55.0)	39 (4.6)	0	11 (34.4)
Comorbid conditions					
Any	319 (26.3)	65 (20.3)	241 (28.2)	4 (80.0)	9 (28.1)
Diabetes	60 (5.0)	22 (6.9)	36 (4.2)	0	2 (6.3)
Hypertension	75 (6.2)	11(3.4)	57 (6.7)	0	2 (0.2)
Asthma	33 (2.7)	5 (1.6)	28 (3.3)	0	0
Heart disease	13 (1.1)	5 (1.6)	8 (0.9)	0	0
PWH	110 (9.1)	18 (5.6)	83 (9.7)	3 (60.0)	4 (18.8)
CD4 cell count, median (IQR), cells/μL	396 (213–610)	360 (89–416)	434 (240–631)	379 (NA)	616 (157–667)
Tobacco smoking	507 (41.9)	140 (43.8)	350 (40.9)	2 (40.0)	15 (46.9)
Duration of tobacco smoking, median (IQR), y	14 (7–20)	14 (6.3–20)	14 (6–20)	11.5 (NA)	14 (9–19)
Inhaled drug use	152 (13.6)	45 (14.1)	97 (11.4)	1 (20.0)	6 (18.8)
Other respiratory pathogens					
Any pathogen^c^	487 (40.2)	101 (31.6)	372 (43.6)	2 (40.0)	12 (37.5)
Virus^d^	294 (24.3)	67 (21.4)	220 (25.8)	0	7 (21.9)
Bacteria	282 (23.3)	51 (15.9)	218 (25.5)	2 (40.0)	11 (34.4)
SARS-CoV-2 (PCR)	114 (9.4)	23 (7.2)	86 (10.1)	0	5 (15.6)
SARS-CoV-2 (antibody)^e^	372 (65.7)	92 (67.6)	265 (65.4)	1 (33.3)	14 (63.6)

Abbreviations: BMI, body mass index; IQR, interquartile range; NA, not applicable (too few to calculate IQR); PCR, polymerase chain reaction; PWH, people with human immunodeficiency virus; SARS-CoV-2, severe acute respiratory syndrome coronavirus 2.

^a^Data represent no. (%) of participants unless otherwise specified.

^b^BMI calculated as weight in kilograms divided by height in meters squared

^c^In some participants, nasopharyngeal swab sample PCR demonstrated >1 pathogen (full list in Supplementary Table 4).

^d^Including SARS-CoV-2.

^e^Only a subset of 566 participants were tested.

“Definite tuberculosis” and “probable tuberculosis” were combined into a “tuberculosis-positive” group for analysis. Overall, 320 (27.1%) were tuberculosis positive (310 with definite and 10 with probable tuberculosis), and 854 (72.4%) were tuberculosis negative. Alternate diagnoses in the “tuberculosis-negative” group and details of the tuberculosis-positive group are in Supplementary Tables 2 and 3. Of the enrolled participants, 501 (41.4%) were female, the median (interquartile range) age was 34 (25.9–45.1) years, with only 31 (2.6%) participants <18 years. Of these participants, 319 (26.3%) had a comorbid illness, of whom 110 (9.1%) were with HIV (with a median [interquartile range] CD4 cell count of 396/μL [213–610/μL]), and 61 (5%) were diabetic. Twenty-nine (9.1%) in the tuberculosis-positive group had prior tuberculosis, compared with 143 (16.7%) in the tuberculosis-negative group. A higher proportion of participants in the tuberculosis-negative group tested PCR positive for another respiratory pathogen (43.6% vs 31.6% in the tuberculosis-positive group), and most of this difference was attributable to bacteria rather than viruses.

### Performance of the Xpert-HR

**Figure 2.**
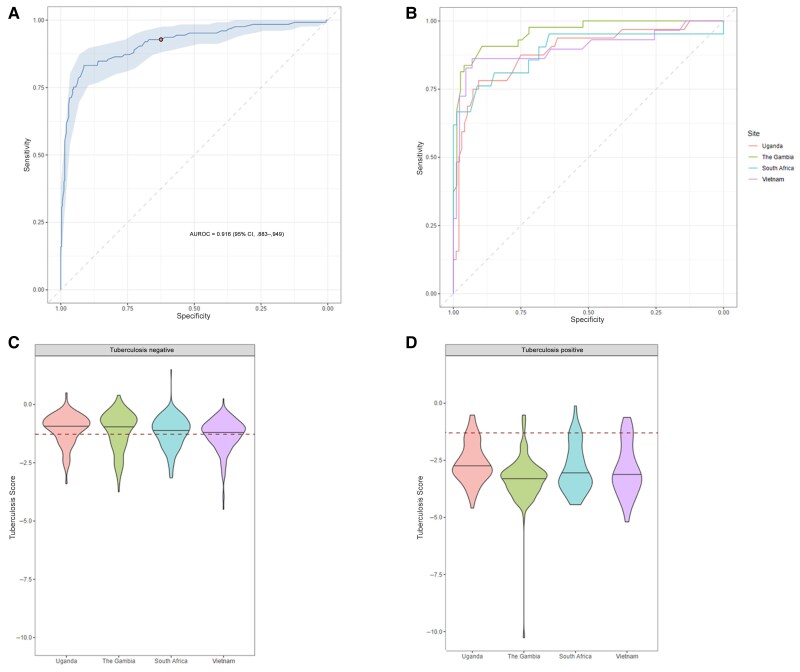
Performance of the Xpert MTB Host Response assay (Xpert-HR). *A*, Area under the receiver operating characteristic (ROC) curve (AUROC) curve for the Xpert-HR TB score (using *TBP* in place of *KLF2*) against the composite reference standard (CRS). *B*, ROC curves for the Xpert-HR TB score at different study sites. *C, D,* Comparative median TB scores in participants classified as “tuberculosis positive” (*C*) or “tuberculosis negative” (*D*) by the CRS, at different study sites. Results of independent median tests were significant for comparisons of Uganda versus Vietnam (*P* < .001), Uganda versus South Africa (*P* = .03), and The Gambia versus Vietnam (*P* = .004) in the tuberculosis-negative group and for Uganda versus The Gambia (*P* < .001) in the tuberculosis-positive group. Dashed red lines represent the optimal cutoff between tuberculosis-positive and tuberculosis-negative classifications in this analysis. Abbreviation: CI, confidence interval.

**Figure 3.**
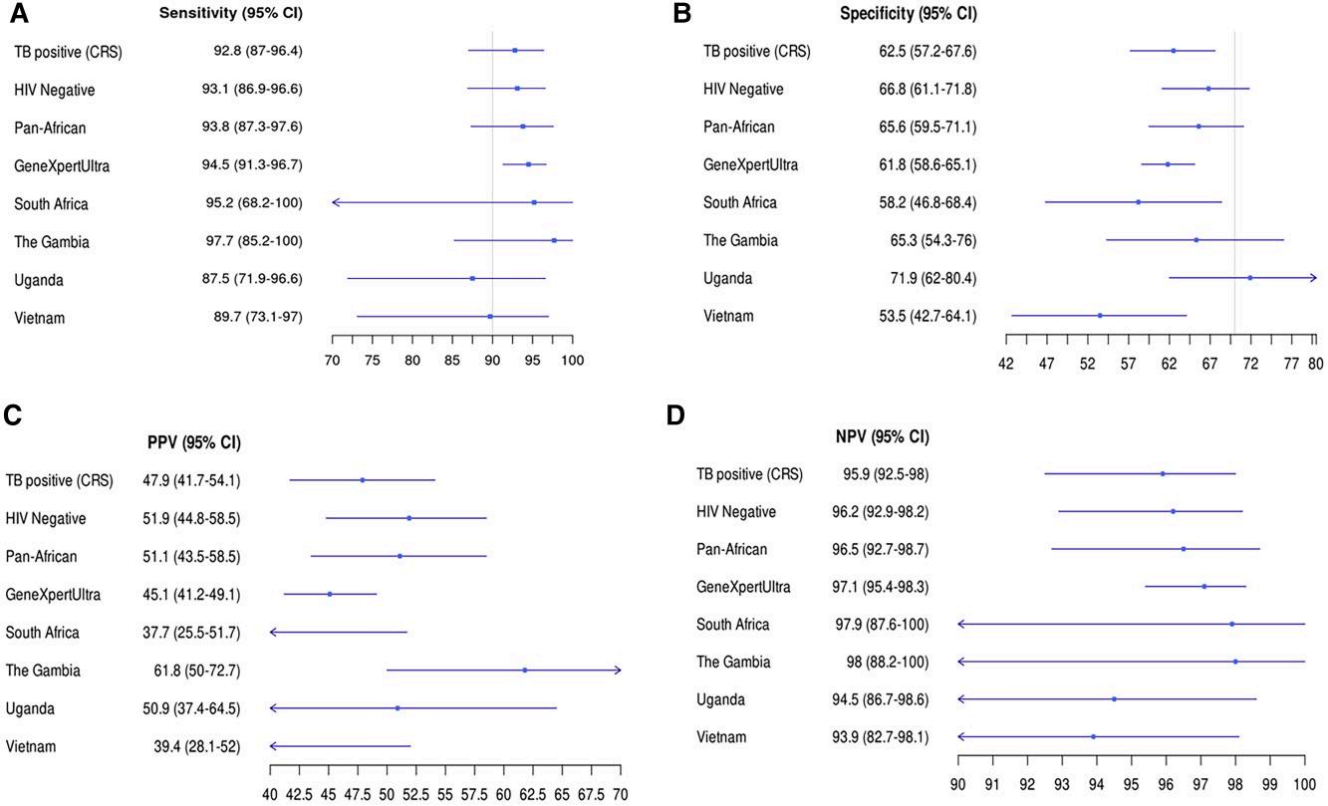
Forest plots comparing the measures of diagnostic accuracy for the Xpert MTB Host Response assay (Xpert-HR) between subgroups and sites. *A*, Sensitivity. *B*, Specificity. *C*, Positive predictive value (PPV). *D,* Negative predictive value (NPV). Vertical lines represent World Health Organization target product profile cutoffs of ≥90% sensitivity and ≥70% specificity. Abbreviations: CI, confidence interval; CRS, composite reference standard; HIV, human immunodeficiency virus.

At an optimal cutoff of −1.275 or below, the TB score was able to discriminate between tuberculosis positive and tuberculosis negative in the CRS, with an AUROC of 0.92 (95% CI: .88%–.95%) (Figure 2*A*), a sensitivity of 92.8% (87.0%–96.4%), a specificity of 62.5% (57.1%–67.6%), a positive predictive value (PPV) of 47.9% (41.6%–54.2%), and a negative predictive value (NPV) of 95.9% (92.4%–98.0%). Performance varied between sites, with the best sensitivity obtained in The Gambia and the poorest in Uganda (Figure 2*B*). The median TB score different between sites, with Uganda having the highest scores in both tuberculosis-positive and tuberculosis-negative groups (Figure 2*C* and 2*D*). Results were similar with an Xpert Ultra reference standard, with an AUROC of 0.92 (95% CI: .89–.95) (Figure 3). Performance was similar in the HIV-negative group and in the pan-African group (excluding Vietnam) (Figure 3). In people with HIV, Xpert-HR had an AUROC of 0.77 (95% CI: .54–.99) (Supplementary Figure 1). Using the original 3-gene signature with *KLF2* resulted in slightly poorer performance, with an optimal cutoff of ≤2.8 yielding an AUROC of 0.91 (95% CI: .87–.94), a sensitivity of 92.0% (86.1%–95.9%), a specificity of 60.7% (55.3%–66%), a PPV of 46.6% (40.5%–52.8%), and NPV of 95.3% (91.7%–97.7%) (Supplementary Figure 1*D*).

We also assessed the performance of Xpert-HR using the previously identified cutoff of −1.25 or below on all 1154 of our results [5]. Using this cutoff, Xpert-HR yielded an AUROC of 0.91 (95% CI: .89–.93), with a sensitivity of 93.0% (89.9%–95.4%), a specificity of 61.4% (58%–64.7%), a PPV of 47.2% (43.3%–51.1%) and an NPV of 95.9% (94.1%–97.3%) (Supplementary Figure 1*E*). The detection of another respiratory pathogen on nasopharyngeal swab sample did not have any significant confounding effect on the Xpert-HR test result (Supplementary Figure 1*F*). Xpert-HR classified as tuberculosis positive 3 of 4 (75%) participants with possible tuberculosis and 18 of 30 (60.0%) who were “unclassifiable” with the CRS.

### Performance of the MBT

**Figure 4.**
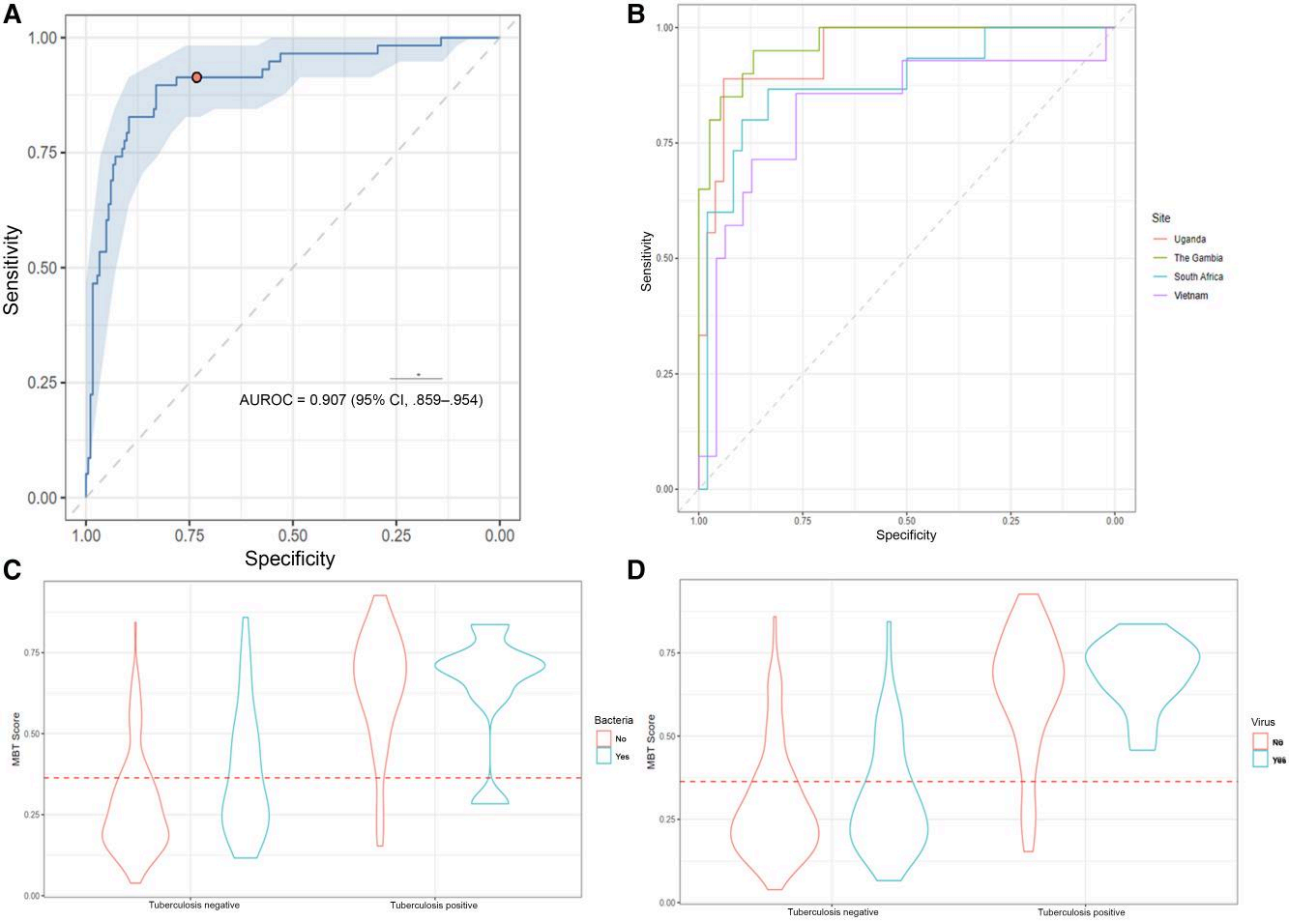
Performance of the multibiomarker test (MBT). *A*, Receiver operating characteristic (ROC) curve for the MBT against the composite reference standard. *B*, ROC curves for the MBT at different study sites. *C*, Effect of bacteria detectable with nasopharyngeal swab sample polymerase chain reaction (PCR) on the MBT (*P* = .004 for the tuberculosis-negative group). *D*, Effect of a virus detectable with nasopharyngeal swab sample PCR on the MBT. Dashed red lines represent the optimal cutoff between “tuberculosis positive” and “tuberculosis negative” in this analysis. Abbreviations: AUROC, area under the ROC; CI, confidence interval.

**Figure 5.**
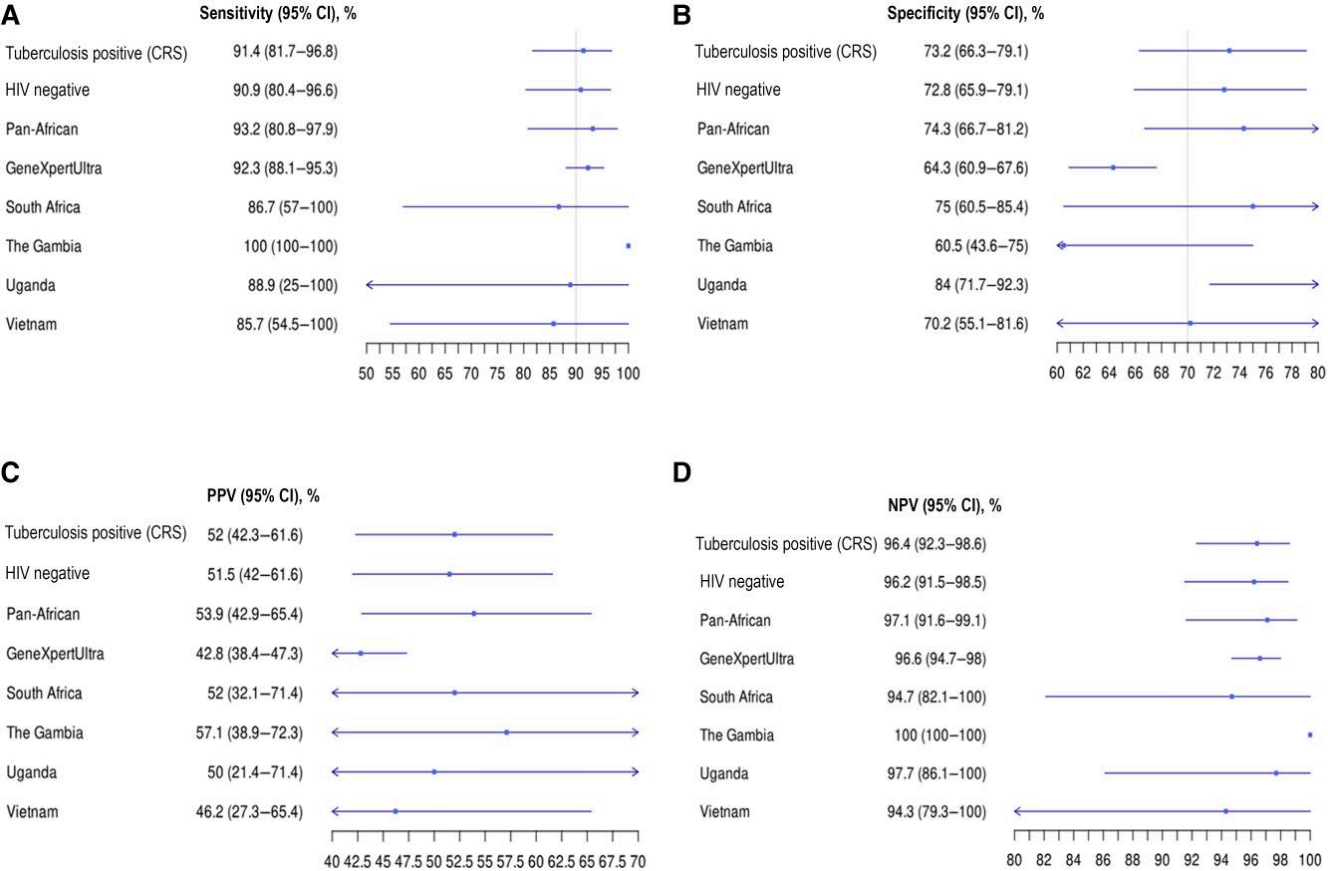
Forest plots comparing the measures of diagnostic accuracy of the multibiomarker test (MBT) between subgroups and sites. *A*, Sensitivity. *B*, Specificity. *C*, Positive predictive value (PPV). *D,* Negative predictive value (NPV). Vertical lines represent World Health Organization target product profile cutoffs of ≥90% sensitivity and ≥70% specificity. Abbreviations: CI, confidence interval; CRS, composite reference standard; HIV, human immunodeficiency virus.

The MBT was able to discriminate between tuberculosis positive and tuberculosis negative in the CRS with an AUROC of 0.91 (95% CI: .86–.96), a sensitivity of 91.4% (81.2%–96.7%), a specificity of 73.2% (66.1%–79.3%), a PPV of 52.0% (42.2%–61.5%), and an NPV of 96.4% (91.8%–98.6%) at a cutoff of ≥0.36 (Figure 4*A*). There was similar variation in performance between sites with this test, with the worst sensitivity in participants from Vietnam (Figure 4*B*).The MBT performed well when compared to an Xpert Ultra reference standard, with a sensitivity of 92.3% (95% CI: 88.1%–95.4%) and an NPV of 96.6% (94.7%–97.9%); however, the specificity dropped to 64.3% (60.9%–67.6%) (Figure 5). Performance was similar in the HIV-negative group and in the pan-African group (Figure 5). In people with HIV, the MBT had an AUROC of 0.90 (95% CI: .66–1.00) (Supplementary Figure 2). Participants with bacteria on nasopharyngeal swab sample PCR had a 9% higher likelihood of a false-positive MBT result for tuberculosis in the absence of tuberculosis disease (*P* = .004), as shown in Figure 4*C*. Viral coinfection did not affect the test outcome, including SARS-CoV-2 (figure 4*D*). MBT classified as tuberculosis positive 1 of 4 participants (25.0%) with possible tuberculosis and 12 of 26 (46.2%) who were unclassifiable with the CRS.

## DISCUSSION

We report a head-to-head prospective evaluation of 2 finger-stick blood tests for triage of people presenting with symptoms suggestive of tuberculosis in a large, multisite study. Both tests performed well, with an AUROC of 0.92 for Xpert-HR and 0.91 for MBT when evaluated against a CRS, but only the MBT reached a specificity of >70%, the minimal requirement of the WHO TPP. However, while Xpert-HR was not affected by the presence of another respiratory pathogen, the MBT had a slightly higher likelihood of yielding a false-positive for tuberculosis in the presence of other bacteria. Importantly, we found a very similar cutoff for the Xpert-HR test, as previously found in another large multisite study [5]. The good overall performance of both tests shows the potential for screening of people presenting with respiratory symptoms at a primary care level. It will be important to see how these findings translate to individuals who are unable to produce sputum, people with HIV, those with extrapulmonary tuberculosis, those with asymptomatic tuberculosis, and young children—an analysis of the latter is currently underway in both The Gambia and Vietnam. It is also important to explore whether additional blood or imaging biomarkers reduce the number of MBT false-positives caused by bacterial infection.

The slight increase in specificity when only African participants were included in the analysis—despite these sites having a higher proportion of people with HIV and previous tuberculosis than the non-African site—is likely due to higher disease severity, as shown by the proportion of participants with tuberculosis who have cavitary disease. Moreover, as seen in several previous studies, more severe disease in general is correlated with an increase in blood-derived inflammatory signals, with better classification seen in people with higher Xpert Ultra readings [9, 15]. This suggests that both tests could be investigated for their use in monitoring of treatment response, as done previously with the MBT [9].

Interestingly, although all other sites showed better specificity with the MBT than with the Xpert-HR, the Gambian site yielded better specificity with the Xpert-HR. This may also be attributable to disease severity. While host genetics should not play a role in variability between the 2 tests, the higher number of participants with sputum Xpert Ultra “high” grading and smear 3+ ranking (shown in Supplementary Table 3) likely indicates a longer time to treatment initiation and more severe disease at diagnosis in The Gambia than at the other sites. Higher levels of host inflammatory markers may influence the performance of the messenger RNA (mRNA), and the protein-based tests differently since production of mRNA and proteins are subject to different kinetics and detection methods.

Major strengths of our study included the evaluation of the tests at primary clinics on people presenting with respiratory symptoms but before any diagnosis and the use of quantified volumes of capillary blood from a finger-stick for both tests, as this procedure is simple and requires minimal training. The good performance of the MBT with capillary blood is especially promising for translation to remote settings, where tuberculosis diagnosis is delayed and costly.

In future, cost, infrastructure requirements, and test setting will guide how each test is used in the diagnostic algorithm. The Xpert-HR, though it has a quick turnaround time, needs a laboratory. Cost of the instrument, cost of the cartridge, and the distance between the instrument and the point of care, must be considered for cost-effectiveness calculations. MBT might be more suitable for widespread use, as the lateral flow technology lends itself to bedside use, and cassettes will likely cost less to manufacture. It is important to remember that these tests were evaluated against the TPP for a triage test, which means that people who test positive still require further confirmatory testing before starting treatment. However, similar to previous reports on the Xpert-HR [5], the high NPV of both tests suggest they will be useful as rule-out tests, to reduce the number of confirmatory tests needed. A point-of-care blood test that can rule out tuberculosis in 70% of the symptomatic population is preferable to a sputum-based molecular test with a higher cost and longer turnaround time.

The differences in stage of development of the 2 tests in this study is one of the study’s limitations. Even though our results suggest a higher diagnostic accuracy and smaller percentage of inactionable results with the MBT compared with the Xpert-HR (0.2% vs 3.7%, respectively), the MBT will have to be assessed in larger prospective studies with a final, design-locked version of the lateral flow cassette, similar to that used for *Mycobacterium leprae* [16, 17]. In addition, the number of people with HIV in our study was too small (110 [9.1%]) for analysis.

In conclusion, we show the potential for 2 novel quantitative point-of-care assays using finger-stick blood as triage tests for symptomatic pulmonary tuberculosis. That the mRNA and protein-based assay yielded similar sensitivities is relevant to the field and will allow flexibility in diagnostic pathways between different tiers of the healthcare system. Importantly, both tests provide rapid sample-to-result time and do not require extensively trained personnel. Same-day test results will allow immediate and appropriate referrals for confirmatory testing and narrow the gaps in the tuberculosis care cascade.

## Supplementary Material

ciaf105_Supplementary_Data

## Supplementary Data

Supplementary materials are available at *Clinical Infectious Diseases* online. Consisting of data provided by the authors to benefit the reader, the posted materials are not copyedited and are the sole responsibility of the authors, so questions or comments should be addressed to the corresponding author.

## Notes

***Acknowledgments***. The authors first acknowledge all of the participants and their families. They also acknowledge the contribution of all TrENDx-TB consortium members: Medical Research Council Unit The Gambia—J. S. S., O. O., Amie Secka, Bintou Njai, Abdou K. Sillah, Georgetta K. Daffeh, A. Gindeh, Amadou Barry, Momodou Rashid, Joseph Mendy, Binta Sarr, Abi-Janet Riley, Alhaji Jobe, Monica Davies, Kairaba Kanyi, Momodou Jallow, Salieu Barry, and Ousainou Cham; Stellenbosch University South Africa—G. W., S. T. M., B. S., G. D. v. d. S., K. S., J. A. S., Alicia Chetram, T. R., Bernadine Fransman, Isaac Johnson, Marika Finn, Andriette Hiemstra, N. N. C., Helena Kuivaniemi, Gerard Tromp, Susanne Tonsing, Elizma Smit, and Balie Carstens; Makerere Uganda—H. M. K., M. N., A. N., S. N., Joseph Akol, Saidah Menya, Veronica Kizza, Yusuf Kironde, Deborah Banturaki, Immaculate Nahereza, Simon Okiror, Immaculate Kemigisha, Paul Mutumba, Henry Ojiambo, Lilian Murungi, Joan Nassuna, Gladys Mpalanyi, and Michael Odie; Oxford University Clinical Research Unit Vietnam—G. T., N. T. T. T., Van Le, Son Vo Thanh, Hau Nguyen Thi, Ha Vu Thi Ngoc, and Ngoc Le Hong; CSU—J. B. and Karen Dobos; LSHTM—H. M. D.; South African Tuberculosis Vaccine Initiative—T. J. S., Mark Hatherill, Kate Hadley, Justin Shenje, Stanley Kimbung, Humphrey Mulenga, Rachel Oelofse, Nicole Bilek; Elma van Rooyen, and Simba Mabwe; Leiden University Medical Centre—P. C., A. Geluk, E. M. T. K. F., Louise Pierneef, and Anouk van Hooij; Catalysis—J. W.; FIND—Morten Ruhwald, E. M., and A. P. N.; and LINQ—Claudia Schacht and Julia Büech.

***Author contributions*.** J. S. S., A. Geluk., P. C., and G. W. conceived and designed the study. J. S. S., T. N. T. T., H. M. K., J. W., H. M. D., T. J. S., A. Geluk., P. C., and G. W. obtained funding. J. A. S., T. R., E. M. T. K. F., A. Gindeh., O. O., M. N., A. N., S. N., B. S., S. T. M., and P. C. developed protocols, recruited participants, and collected data. N. N. C. contributed to study design and the interpretation of results. G. D. v. d. S., T. R., and K. S. designed the database. J. W. and B. S. provided project support. G. D. v. d. S. led the power calculations, statistical analysis, and interpretation. J. S. S. and J. A. S. wrote the original manuscript, and all authors reviewed and edited manuscript drafts. J. S. S., G. D. v. d. S., T. R., E. M. T. K. F., K. S., A. Geluk., S. T. M., P. C., and G. W. verified the underlying data.

***Financial support.*** This work was supported by European & Developing Countries Clinical Trials Partnership (grant RIA2018D-2499; principal investigator, G. W.), the National Institutes of Health (grant U01AI152075; principal investigators, J. S. S., J. B., and G. W.), the Bill & Melinda Gates Foundation (grant INV-023825), the South African Medical Research Council (support to the Stellenbosch University participants as part of the Centre for Tuberculosis Research), and the South African National Research Foundation (grant SANRF86535 to G. W.).

Under the grant conditions of the Bill & Melinda Gates Foundation, a Creative Commons Attribution 4.0 Generic License has already been assigned to the author accepted manuscript version that might arise from this submission.

***Data sharing*.** Data collected for the study, including individual participant data and a data dictionary defining each field in the set, are available at https://zenodo.org/records/11108773.
