# Supplementary material for: Performance of 2 Finger-Stick Blood Tests to Triage Adults With Symptoms of Pulmonary Tuberculosis: A Prospective Multisite Diagnostic Accuracy Study
Source: Clin Infect Dis. 2025 Apr 16;81(4):857–66. doi: 10.1093/cid/ciaf105 (PMC12596368; doi:10.1093/cid/ciaf105)
Supplement: ciaf105_Supplementary_Data [file ciaf105_supplementary_data.pdf]

Performance of two fingerstick blood tests to triage adults with symptoms of pulmonary TB: a prospective multisite diagnostic accuracy study

Table of contents

1. Supplementary methods
    1.1. Sample size calculation
    1.2. HIV testing
    1.3. Other respiratory pathogens PCR
    1.4. Anti-SARS_CoV-2 antibody testing
    1.5. Xpert-HR
    1.6. Production and assay protocol of the Multibiomarker test (MBT)

2. Supplementary tables
    Table 1: Characteristics of the participants by site
    Table 2: Alternate diagnoses in the TB negative group
    Table 3: Details of the TB positive group
    Table 4: Other respiratory pathogens identified on PCR

3. Supplementary figures
    Figure 1: Performance of the Xpert-HR TB Score
    Figure 2: Performance of the MBT

1. Supplementary methods

1.1 Sample size calculation

Sample size calculations were performed for the validation of the MBT as described in the published trial protocol. The required endpoints are sensitivity, specificity, positive and negative predictive value with 95% CI, with half-width dependent on sample size. From previous experience, 80 participants per site were expected to have active TB, or one third of the total enrolled sample (or 333/1000), resulting in a target sensitivity of 90% with a 95% CI half-width of 5%. Thus, target precision should be achieved for analysis of MBT performance at each site and overall.

1.2 HIV testing

All participants without known HIV infection received voluntary counselling and testing using a rapid fingerstick blood HIV-1/2 test (Alere, USA). If the rapid test was positive, the result was confirmed with a second rapid test (RightSign HIV, Hangzhou Biotest Biotech Ltd). If these were discordant, a serum sample was submitted to the reference laboratory for HIV ELISA. CD4 counts were determined for all HIV+ individuals at baseline.

1.3 Other respiratory pathogens PCR

Nasopharyngeal swabs were collected in UTM for immediate processing with in-house SARS-CoV-2 PCR testing or GeneXpert SARS-CoV-2 Xpert Xpress cartridges (Cepheid, Sunnyvale, CA, USA). For the GeneXpert SARS-CoV-2 Xpress cartridges (Cepheid, USA), samples were loaded onto a GeneXpert SARS-CoV-2 Xpress cartridge using a transfer pipette. The cartridge was then inserted into the GeneXpert machine and run using the GeneXpert Xpress program. Ct values for both the Envelope (E) and Nucleocapsid (N) genes were obtained with a result of 'positive' or 'negative'. Aliquots from processed nasopharyngeal swabs from South Africa and Uganda were stored and shipped to Gambia for a multiplex 26-pathogen PCR analysis (Allplex respiratory panel™, Seegene Inc., Seoul, South Korea). Samples from Vietnam were analysed using the same assay in-country.

1.4 Anti-SARS-CoV-2 antibodies

A subset of participants was tested for anti-SARS-CoV-2 antibodies with Abbott SARS-CoV-2 IgG assay (Abbott Laboratories, South Africa, Pty Ltd) or the Abbott SARS-CoV-2 IgG II Quant (Abbott Laboratories, South Africa, Pty Ltd), both done on the Architect i System (Abbott Laboratories, Illinois, USA). All SARSCoV-2 tests were carried out by the National Health Laboratory Service (NHLS) of South Africa (https:// www.nhls. ac. za/). Participants who were previously vaccinated against SARS-CoV-2 who only had a positive anti-S antibody on serologic testing, and no previous positive RT-qPCR (and therefore no evidence of infection-acquired immunity or previous infection), were not included in the SARS-CoV-2 positive subgroup.

1.5 Xpert-HR

Two hundred (200) µl of capillary blood was collected by fingerstick with a minivette containing an anticoagulant (EDTA, Becton Dickinson, USA) transferred into an EDTA-microtainer (Becton Dickinson, USA), and inverted to mix. 100µl of the sample was added to the Xpert-HR cartridge (donated by Cepheid, USA) and loaded into the GeneXpert machine. Ct values for individual genes (*GBP5, DUSP3, KLF2)* were obtained together with a TB score automatically calculated.[4] If an invalid result was obtained, a second aliquot of 100µl was added to a new cartridge and the sample re-tested. An alternative gene to KLF2 was included by the company (TBP5) with a second TB score generated accordingly to improve stability and performance. The Xpert-HR test evaluates the mRNA levels of the four target genes, *GBP5, DUSP3, KLF2,* and *TBP* by reverse transcriptase PCR (RT-PCR). Cycle threshold values for the individual four genes are obtained. The TB Score is calculated using the following formula: [cycle threshold of *GBP5* + cycle threshold of *DUSP3*)/2 – cycle threshold of *KLF2].* A second score was calculated: [cycle threshold of *GBP5* + cycle threshold of *DUSP3*)/2 – cycle threshold of *TBP5].* No prespecified cutoff value was provided by the manufacturer. If an invalid result was obtained, a second aliquot of 100µl was added to a new cartridge and the sample re-tested.

1.6 Production and assay protocol of the Multi Biomarker Test (MBT):

Lateral flow (LF) strips:

4 mm wide LF strips specific for CRP, IP-10 or SAA1/A2 were produced as described previously.[9] Test (T) lines comprised 200 ng of the following antigen and antibodies: CRP (Labned, The Netherlands), mouse-anti-human IP-10 mAb (B-C55; Diaclone, France) or mouse-anti-human SAA1 mAb (Novus, USA). The downstream Flow-Control (FC) line comprised 100 ng goat-anti-mouse antibody (M8642; Sigma-Aldrich, USA).

UCP Conjugates:
Antibodies were conjugated to luminescent up-converting reporter particles (UCP; 200 nm, $NaYF_4$:Yb^{3+}, Er^{3+}; Intelligent Material Solutions, USA) according to previously described protocols.[9] Mouse-anti-CRP (CRP135; Labned, The Netherlands), mouse-anti-IP-10 (B-C50; Diaclone, France) and mouse-anti-SAA1 (DY3019-05, part 844408, ELISA capture antibody; R&D systems, USA) were bound at a concentration of 50 µg antibody per mg UCP. LF strips were provided with a sample/conjugate pad containing 200 ng of appropriate UCP-conjugate.

MBT procedure:
Fingerstick blood (20µl) was collected using disposable Minivette® collection tubes (Heparin coated; Sarstedt, Germany) and directly mixed with 480µl high salt finger stick (HSFS) buffer: 100 mM Tris pH 8.0, 270 mM NaCl, 1% (v/v) Triton X-100, and 0.5% (w/v) Casein.[7,9] 100µl of the lysed blood was added to a disposable microwell after which lateral flow (LF) strips (SAA1, CRP, IP10) were added. LF was continued until strips were dry after which they were analysed with the ESEQuant (LR3 version) reader adapted for UCP (DI-ALUNOX, Germany). Test results were displayed as the ratio (R=T/FC) between Test and Flow-Control signal.

2. Supplementary tables

Table 1: Characteristics of the participants by site

	**South Africa (n=305)**	**The Gambia (n=301)**	**Uganda (n=304)**	**Vietnam (n=301)**
**Females**	146 (47.9)	116 (38.5)	85 (28.0)	155 (51.5)
**Age**	38.1 [28.2-47.9]	29.8 [23-40.3]	31.4 [25.3-39.2]	37.4 [27.9-51.2]
**Previous TB**	126 (41.3)	14 (4.7)	23 (7.6)	21 (7.0)
**BMI**	19.8 [18.1-24.3]	19 [17.1-21.8]	20.3 [18.2-22.3]	20.8 [18.9-23.3]
**TB positive**	51 (16.7)	105 (34.9)	75 (24.7)	89 (29.6)
**PLWHIV**	38 (12.5)	14 (4.7)	51 (16.8)	5 (1.7)
**Tobacco smoking**	249 (81.6)	72 (23.9)	146 (48.0)	77 (25.6)
**Inhaled drug use**	71 (23.3)	25 (8.3)	50 (16.4)	3 (1.0)
**CXR cavities**	59 (19.3)	67 (22.3)	61 (20.0)	34 (11.3)

TB, tuberculosis; BMI, body mass index; PLWHIV, people living with HIV, CXR, chest X-ray.

Table 2: Alternate diagnoses in the TB negative group

**Diagnosis (group)**	n**, %**
Other respiratory tract infection or sequelae	330 (38.6)
Asthma or other allergic respiratory tract disease	134 (15.7)
Post-Tuberculosis Lung Disease	90 (10.5)
Smoking-related lung disease including COPD	42 (4.9)
Gastroesophageal reflux disease or gastritis	20 (2.3)
Other (e.g. heart disease, cancer)	25 (2.9)
No specific alternative diagnosis made, but TB tests negative and symptoms resolved on 8-week follow-up	150 (17.6)
All investigations negative for TB, no week 8 follow up	63 (7.4)
**Total**	854

COPD, chronic obstructive pulmonary disease; TB, tuberculosis

Table 3: Details of the TB positive group

	**All (n=320)**	**The Gambia** (n=105)	**South Africa** (n=51)	**Vietnam** (n=89)	**Uganda** (n=75)
**Xpert Ultra ranking,** n **(%)**					
**High**	131 (40.9)	59 (56.2)	17 (33.3)	27 (30.3)	28 (37.3)
**Medium**	76 (23.8)	26 (24.8)	11 (21.6)	20 (22.5)	19 (25.3)
**Low**	56 (17.5)	9 (8.6)	13 (25.5)	21 (23.6)	13 (17.3)
**Very low**	21 (6.6)	4 (3.8)	2 (9.5)	8 (8.9)	7 (9.3)
**Trace**	12 (3.4)	0	2 (9.5)	7 (7.9)	3 (4.0)
**Unavailable**	5 (1.6)	0	5 (9.8)	0	0
**Negative**	19 (5.0)	7 (6.7)	1 (1.9)	6 (6.7)	5 (6.7)
**Smear grade,** n **(%)**					
**3+**	82 (25.6)	41 (39.1)	5 (9.8)	11 (12.4)	25 (33.3)
**2+**	64 (20.0)	23 (21.9)	7 (13.7)	14 (15.7)	20 (26.7)
**1+**	74 (23.1)	26 (24.8)	11 (21.6)	33 (37.1)	4 (5.3)
**<10 per all fields**	28 (8.8)	0	3 (5.9)	24 (26.9)	0
**Negative**	70 (21.9)	15 (14.3)	24 (47.1)	6 (6.7)	15 (20.0)
**MGIT Culture**, n (%)					
**Positive**	278 (86.7)	93 (88.6)	45 (88.2)	76 (85.3)	64 (85.3)
**TTP days (median, [IQR])**	7 [5-11]	7 [5-9]	5 [4-8]	8 [6-15]	8 [5.3-10]
**Chest X-ray findings,** n **(%)**					
**Cavitation**	176 (55.0)	63 (60.0)	31 (60.8)	32 (35.6)	50 (66.7)
**Pleural effusion**	27 (8.4)	6 (5.7)	4 (7.8)	6 (6.7)	8 (10.7)
**Consolidation**	163 (50.9)	33 (31.4)	18 (35.3)	17 (19.1)	33 (44.0)
**Infiltrates**	270 (84.4)	93 (88.6)	45 (88.2)	65 (73.0)	67 (89.3)
**Nodules**	47 (14.7)	1 (1.0)	8 (15.7)	0	38 (50.7)
**Lymphadenopathy**	6 (1.9)	0	2 (3.9)	0	4 (5.3)

IQR, interquartile range; MGIT, mycobacterial growth inhibition tube; TTP, time to positivity.

Table 4: Other respiratory pathogens identified on PCR

	All*	TB positive	TB negative	Possible TB	Unclassifiable
Influenza A virus	7	1	6	0	0
Influenza B virus	5	1	4	0	0
Respiratory Syncitial Virus	9	1	7	0	1
Adenovirus	4	1	3	0	0
Enterovirus	6	2	4	0	0
Metapneumovirus	6	0	6	0	0
Parainfluenza virus (1-4)	12	5	7	0	0
Bocavirus	7	2	5	0	0
SARS-CoV-2	114	23	86	0	5
Other coronaviruses (OC43, NL63, 229e)	21	8	12	0	1
Rhinovirus	121	28	92	0	1
Bordatella parapertussis and Bordatella pertussis	0	0	0	0	0
Chlamydophila pneumoniae	6	2	4	0	0
Haemophilus influenzae	186	33	144	1	8
Legionella pneumophila	1	1	0	0	0
Mycoplasma pneumoniae	1	0	1	0	0
Streptococcus pneumoniae	152	23	122	1	6
Total	658	131	503	2	22

*Some participants had more than one pathogen on their nasopharyngeal swab PCR.

3. Supplementary figures

Figure 1: Performance of the Xpert-HR TB Score

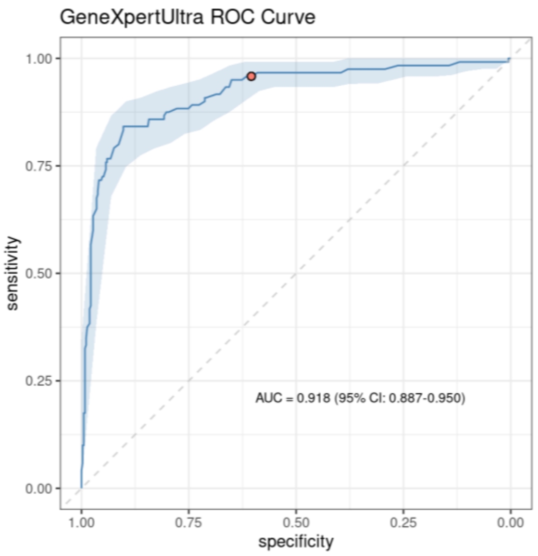

A. Performance of the Xpert-HR TB Score against Xpert Ultra reference standard

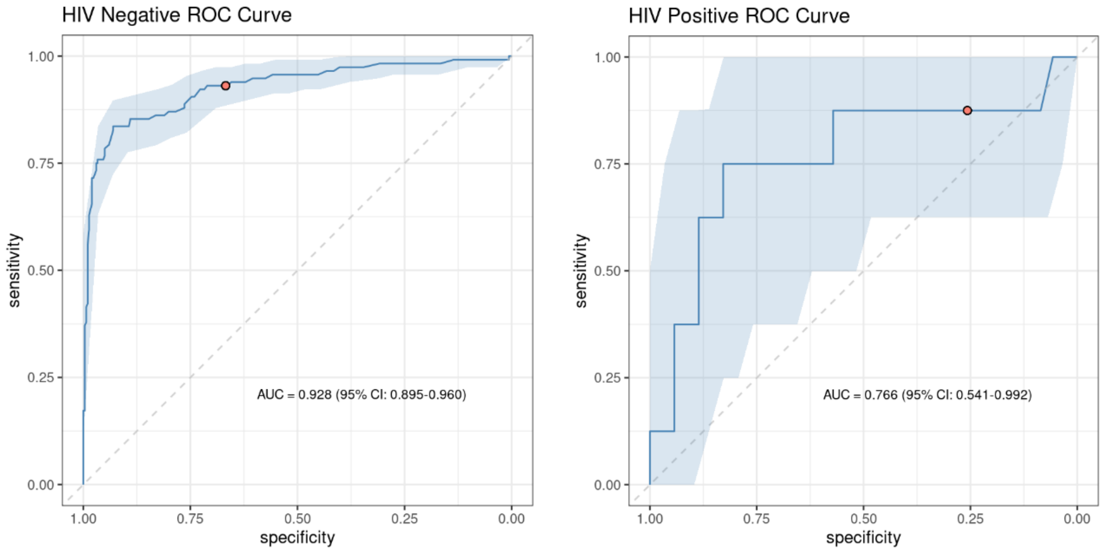

B. Performance of the Xpert-HR TB score according to HIV status

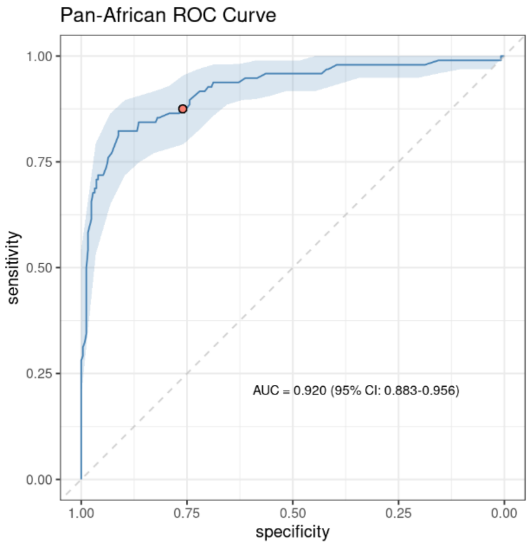

C. Performance of the Xpert-HR TB score in the Pan-African cohort

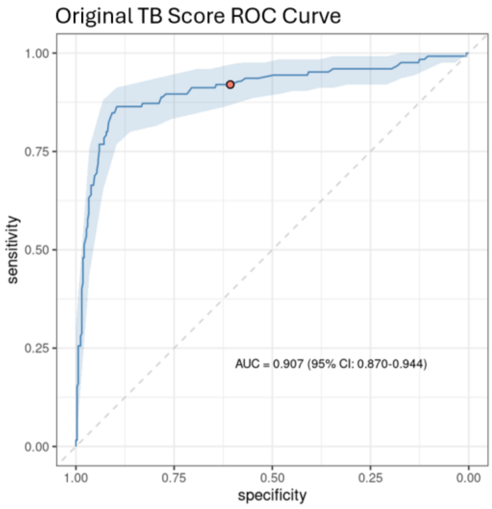

D. Performance of the original Xpert-HR TB Score with KLF2 in place of TBP

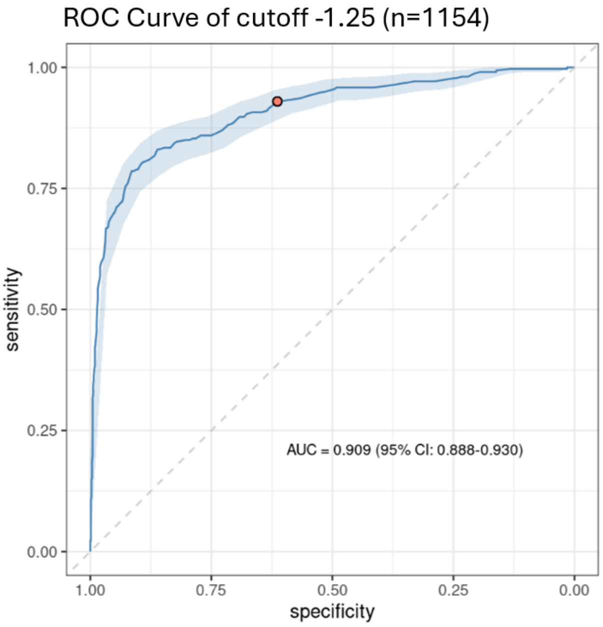

Full Data-set Performance at Pre-defined Threshold (-1.25)					
	Positive n	Sensitivity	Specificity	PPV	NPV
**TB positive (CRS)**	616	93 (89.9-95.4)	61.4 (58-64.7)	47.2 (43.3-51.1)	95.9 (94.1-97.3)

E. Performance of the cutoff of ≤-1.25 identified by Gupta-Wright et al (Lancet Glob Health 2024;12: e226–34) on the entire dataset in the present study.

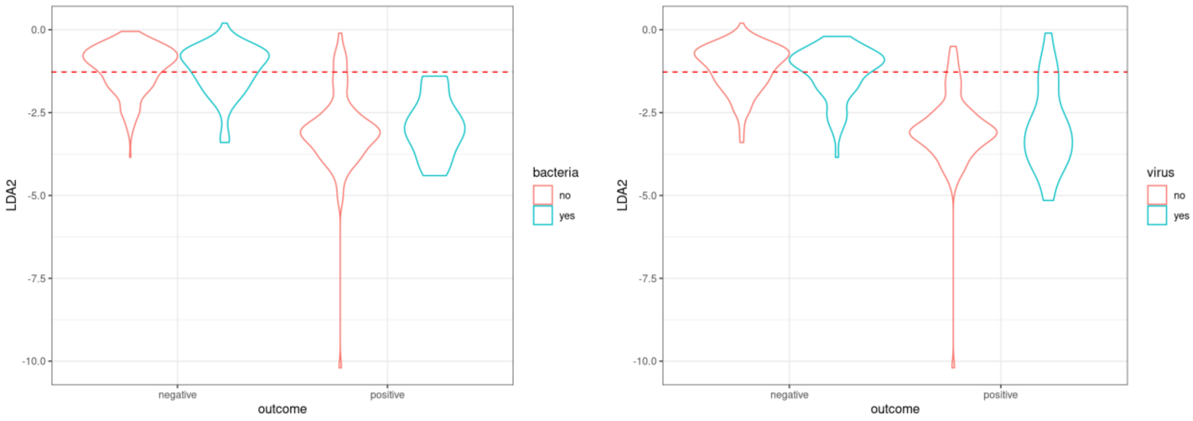

F. Effect of other respiratory pathogens on the performance of the Xpert-HR TB score

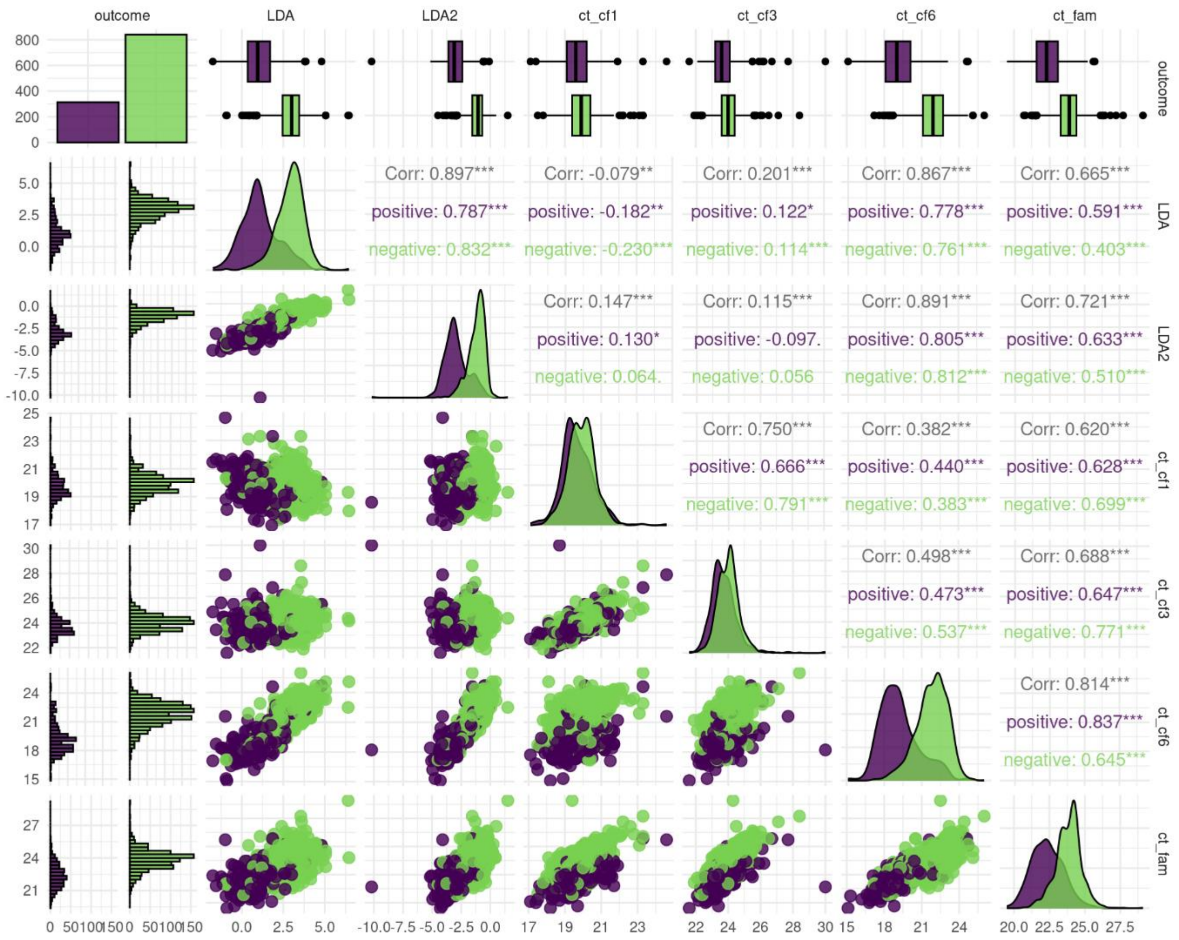

G. Performance of the individual markers of the Xpert-HR TB Score. The figure shows the ability of the Xpert-HR TB scores and individual markers to discriminate between TB positive (purple) and TB negative (green) groups, and the correlations between the markers. LDA, TB score with KLF2 as the third marker (cycle threshold of *GBP5* + cycle threshold of *DUSP3*)/2 – cycle threshold of *KLF2*; LDA2, TB score with TBP as the third marker (cycle threshold of *GBP5* + cycle threshold of *DUSP3*)/2 – cycle threshold of *TBP*; ct_cf1= KLF2 Ct value; ct_cf3 = TBP Ct value; ct_cf6 = GBP5 Ct value; ct_fam = DUSP3 Ct value.

Figure 2: Performance of the MBT

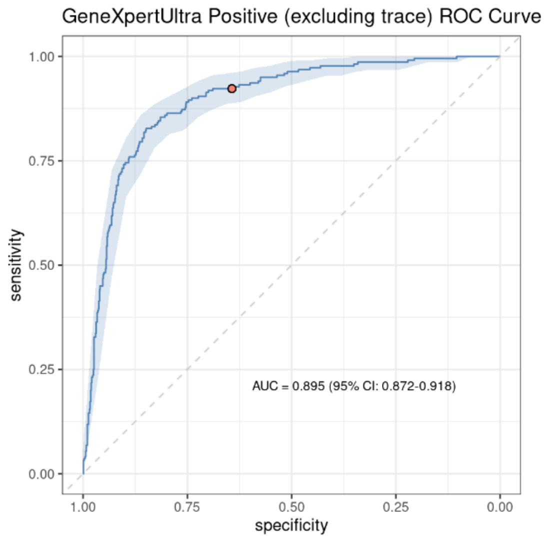

A. Performance of the MBT against Xpert Ultra reference standard

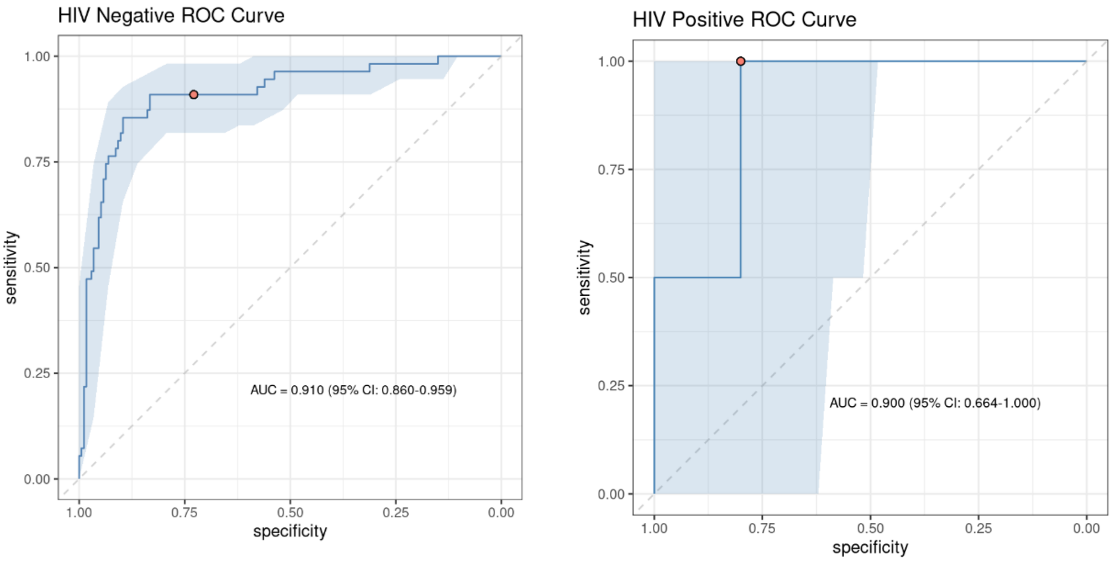

B. Performance of MBT according to HIV status

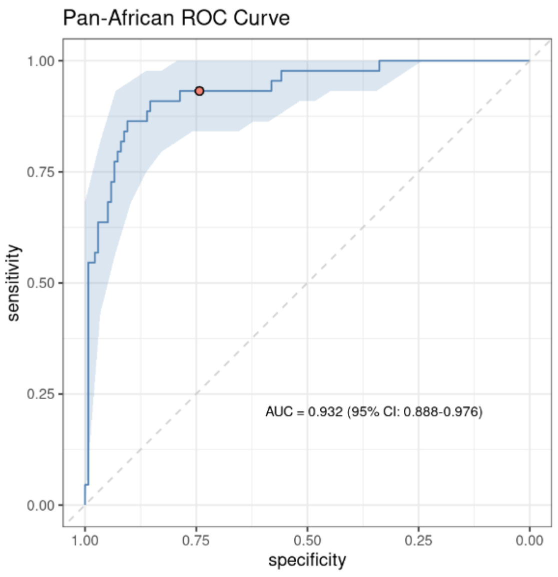

C. Performance of the MBT in the Pan-African cohort

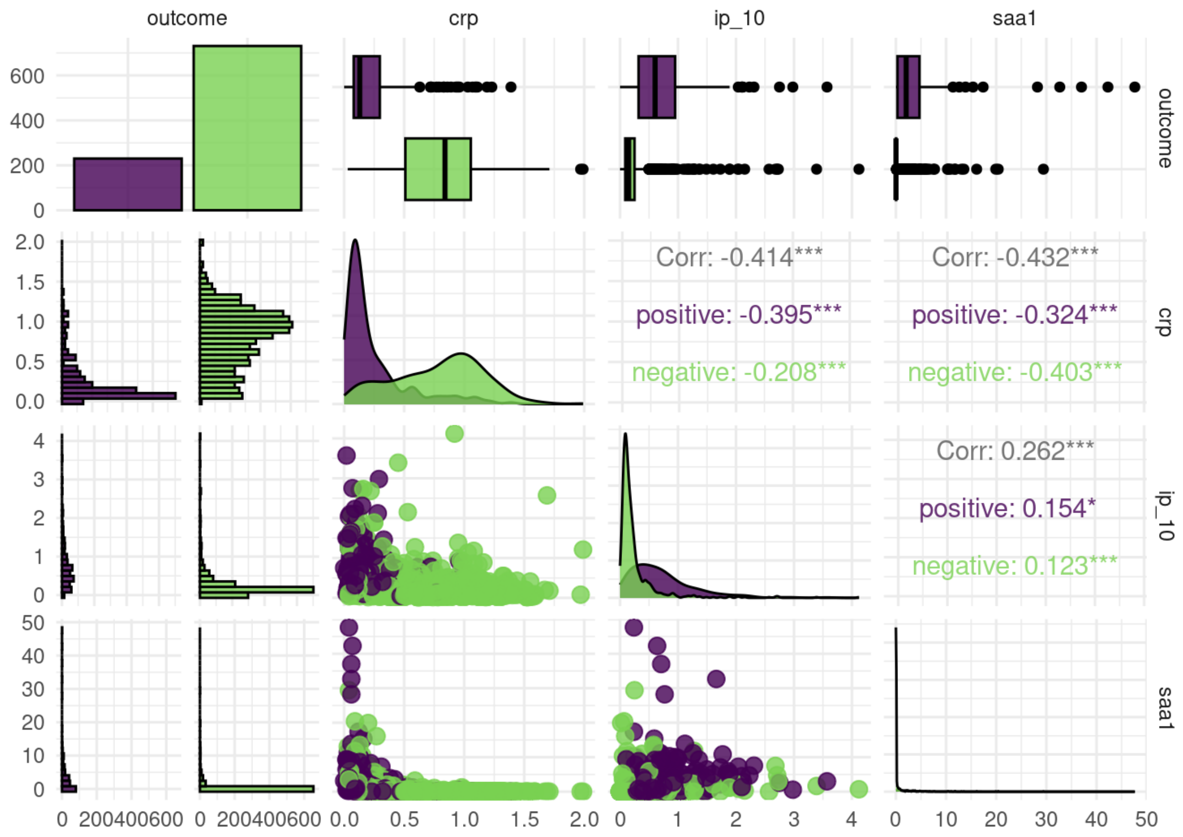

D. Performance of the individual markers in the MBT.

The figure shows the ability of the MBT individual markers to discriminate between TB positive (purple) and TB negative (green) groups, and the correlations between the markers. CRP, C-reactive protein; IP-10, interferon-gamma inducible protein 10; SAA-1, Serum amyloid A-1
